# Draft Whole-Genome Sequence of the Anthracene-Degrading Strain Mycolicibacterium frederiksbergense LB501T, Isolated from a Polycyclic Aromatic Hydrocarbon-Contaminated Soil

**DOI:** 10.1128/MRA.00671-20

**Published:** 2020-10-22

**Authors:** Floriana Augelletti, Julien Tremblay, Spiros N. Agathos, Alexandre Jousset, Ben Stenuit

**Affiliations:** a Earth & Life Institute, Laboratory of Bioengineering, Catholic University of Louvain, Louvain-la-Neuve, Belgium; b Energy, Mining and Environment, National Research Council Canada, Montreal, Quebec, Canada; c Institute of Environmental Biology, Ecology and Biodiversity Group, Utrecht University, Utrecht, The Netherlands; University of Maryland School of Medicine

## Abstract

Here, we report the draft whole-genome sequence of an anthracene-degrading bacterium, Mycolicibacterium frederiksbergense strain LB501T, using the PacBio and Illumina sequencing platforms. The complete genome sequence of strain LB501T consists of 6,713,618 bp and provides new insights into its metabolic capabilities, including aromatic conversion pathways with promiscuous activities.

## ANNOUNCEMENT

Mycolicibacterium frederiksbergense strain LB501T was isolated from a polycyclic aromatic hydrocarbon (PAH)-contaminated soil (Belgium) using PAH-sorbing Teflon membranes (1, 2). Strain LB501T, previously belonging to the genus *Mycobacterium*, has been reclassified into the genus *Mycolicibacterium* (3, 4). The strain was grown in a mineral medium (5) containing 0.5 g liter^−1^ of anthracene as the sole carbon source. DNA was extracted using the DNeasy PowerMax soil kit (Qiagen, Carlsbad, CA) with a modified protocol that included a pretreatment with lysozyme (20 mg ml^−1^, at 37°C for 30 min) and proteinase K (1.8 mg ml^−1^, at 56°C for 30 min) to improve cell lysis. The genome was sequenced using the PacBio RS II (Menlo Park, CA) and Illumina (San Diego, CA) sequencing platforms. Default parameters were used except where otherwise noted. For PacBio sequencing, genomic DNA was directly used for library preparation without fragmentation. A 20-kb SMRTbell library was generated and sequenced on one single-molecule real-time (SMRT) cell at the Génome Québec Innovation Centre (McGill University, Montréal, Canada), which generated 1,238,955,776 bp from 124,389 subreads, with an average subread length of 9,960 bp. Raw subreads shorter than 500 bp or having a quality score lower than 0.75 were filtered out, and the remaining reads were *de novo* assembled using the Hierarchical Genome Assembly Process (HGAP.3)/Quiver protocol from the SMRT Analysis software suite version 2.3.0.140936.p4 (6). The resulting assembly was subsequently scaffolded using SSPACE-Longread version 1.1 (7). To further correct for potential assembly artifacts, paired-end sequencing (2 × 75 bp) was performed on the Illumina NextSeq 500 sequencing platform at the Utrecht Sequencing Facility (Utrecht, The Netherlands). A new DNA extraction was carried out as described above, and a DNA library was prepared using a TruSeq Nano DNA low-throughput library prep kit with a target insert size of 350 bp (Illumina). Illumina raw reads were trimmed using Trimmomatic version 0.32 (palindromic mode, headcrop:16, minlen:32, slidingwindow:4:15, trailing:30), yielding a total number of 5,959,272 (5,959,272 R1 + 5,959,272 R2) Illumina quality-controlled paired-end reads. The Illumina reads were aligned against scaffolds, and a consensus sequence of each scaffold was generated using bcftools version 1.9 (8). The final corrected scaffolds were annotated using the NCBI Prokaryotic Genome Annotation Pipeline (PGAP) version 4.8 (9). The final assembly consists of four scaffolds, with a total size of 6,713,618 bp. One scaffold corresponds to a circular chromosome with a length of 6,086,872 bp and a G+C content of 67.3%. The chromosome was circularized using SSPACE-Longread version 1.1. The other three scaffolds correspond to plasmids, one 341,437-bp plasmid (G+C content, 65.8%), one 268,343-bp plasmid (G+C content, 65.2%), and one 16,966-bp plasmid (G+C content, 66.1%). The chromosome and the 341,437-bp plasmid each contain one within-scaffold gap of 2,142 bp (3,206,107 to 3,208,248) and 491 bp (862 to 1,352), respectively. The whole-genome sequence encompasses 6,440 coding sequences (CDS), 2 rRNA operons (16S, 23S, and 5S rRNAs), 2 noncoding RNAs, and 46 tRNA genes. Two chromosomally encoded catabolic regions of approximately 99 and 33 kb were identified at positions 737,825 to 836,329 and 3,866,266 to 3,899,381, respectively, which contain several gene clusters required for complete PAH biodegradation, such as phenanthrene, anthracene, and pyrene. In particular, the 99-kb catabolic region presents a gene structure and organization similar to those of the 150-kb catabolic region A from Mycobacterium vanbaalenii PYR-1 (10, 11). These regions contain several ring-hydroxylating dioxygenases (RHDs), including *nidAB*, *nidA3B3*, and *pdoA2B2*, responsible for initial dioxygenation in the ring cleavage process of low- and high-molecular-weight PAHs (12, 13). The *pht* cluster (*phtRAaAbBAcAd*) (14) and the β-ketoadipate pathway cluster (*pcaJICDBGHR*) (15, 16) were identified in the major catabolic region, which demonstrates that anthracene degradation by strain LB501T proceeds through the *o*-phthalate degradation pathway (phthalate 3,4-dioxygenase) and *ortho*-cleavage of protocatechuate (protocatechuic acid [PCA] 3,4-dioxygenase), as previously suggested (17). Moreover, the genome sequence harbors multiple genes involved in heavy metal resistance, such as copper (*copA*, *copC*, and *copD*), arsenic (*arsRBCDA*), and mercury (*merA* and *merB*). The presence of these metal resistance genes suggests that *Mycolicibacterium frederiksbergense* strain LB501T could be a promising microorganism for bioaugmentation of soils contaminated with both PAHs and heavy metals.

The genome sequence of strain LB501T will enable transcriptomic and proteomic analyses under different environmental conditions and the development of specific biomarkers for bioaugmentation monitoring. The metabolic capabilities of strain LB501T are also relevant to biorefining processes of lignin fractions of lignocellulosic biomass (i.e., the bioaromatic platform) (18, 19).

### Data availability.

This whole-genome shotgun project has been deposited at GenBank under the BioProject number PRJNA521839, the BioSample number SAMN10915336, and the accession numbers CP038799, CP038797, CP038798, and CP038796. The versions described in this paper are the first versions, CP038799.1, CP038797.1, CP038798.1, and CP038796.1. The PacBio and Illumina raw reads have been deposited in the Sequence Read Archive (SRA) under accession numbers SRR10121029 and SRR11515926, respectively.

## References

[1] Bastiaens L, Springael D, Wattiau P, Harms H, deWachter R, Verachtert H, Diels L 2000 Isolation of adherent polycyclic aromatic hydrocarbon (PAH)-degrading bacteria using PAH-sorbing carriers. Appl Environ Microbiol 66:1834–1843. doi:10.1128/AEM.66.5.1834-1843.2000. 10788347 PMC101420

[2] Wick LY, Pasche N, Bernasconi SM, Pelz O, Harms H 2003 Characterization of multiple-substrate utilization by anthracene-degrading *Mycobacterium frederiksbergense* LB501T. Appl Environ Microbiol 69:6133–6142. doi:10.1128/AEM.69.10.6133-6142.2003. 14532072 PMC201237

[3] Gupta RS, Lo B, Son J 2018 Phylogenomics and comparative genomic studies robustly support division of the genus *Mycobacterium* into an emended genus *Mycobacterium* and four novel genera. Front Microbiol 9:67. doi:10.3389/fmicb.2018.00067. 29497402 PMC5819568

[4] Bachmann NL, Salamzade R, Manson AL, Whittington R, Sintchenko V, Earl AM, Marais BJ 2020 Key transitions in the evolution of rapid and slow growing *Mycobacteria* identified by comparative genomics. Front Microbiol 10:3019. doi:10.3389/fmicb.2019.03019. 32038518 PMC6985099

[5] Schuler L, Ni Chadhain SM, Jouanneau Y, Meyer C, Zylstra GJ, Hols P, Agathos SN 2008 Characterization of a novel angular dioxygenase from fluorene-degrading *Sphingomonas* sp. strain LB126. Appl Environ Microbiol 74:1050–1057. doi:10.1128/AEM.01627-07. 18156320 PMC2258591

[6] Chin C-S, Alexander DH, Marks P, Klammer AA, Drake J, Heiner C, Clum A, Copeland A, Huddleston J, Eichler EE, Turner SW, Korlach J 2013 Nonhybrid, finished microbial genome assemblies from long-read SMRT sequencing data. Nat Methods 10:563–569. doi:10.1038/nmeth.2474. 23644548

[7] Boetzer M, Pirovano W 2014 SSPACE-LongRead: scaffolding bacterial draft genomes using long read sequence information. BMC Bioinformatics 15:211. doi:10.1186/1471-2105-15-211. 24950923 PMC4076250

[8] Li H, Handsaker B, Wysoker A, Fennell T, Ruan J, Homer N, Marth G, Abecasis G, Durbin R, 1000 Genome Project Data Processing Subgroup 2009 The Sequence Alignment/Map format and SAMtools. Bioinformatics 25:2078–2079. doi:10.1093/bioinformatics/btp352. 19505943 PMC2723002

[9] Tatusova T, DiCuccio M, Badretdin A, Chetvernin V, Nawrocki EP, Zaslavsky L, Lomsadze A, Pruitt KD, Borodovsky M, Ostell J 2016 NCBI Prokaryotic Genome Annotation Pipeline. Nucleic Acids Res 44:6614–6624. doi:10.1093/nar/gkw569. 27342282 PMC5001611

[10] Kweon O, Kim S-J, Cerniglia CE 2019 An update on the genomic view of mycobacterial high-molecular-weight polycyclic aromatic hydrocarbon degradation, p 623–638. *In* Rojo F (ed), Aerobic utilization of hydrocarbons, oils, and lipids, 1st ed Springer International Publishing, Cham, Switzerland.

[11] Kim S-J, Kweon O, Jones RC, Edmondson RD, Cerniglia CE 2008 Genomic analysis of polycyclic aromatic hydrocarbon degradation in *Mycobacterium vanbaalenii* PYR-1. Biodegradation 19:859–881. doi:10.1007/s10532-008-9189-z. 18421421

[12] Zeng J, Zhu Q, Wu Y, Chen H, Lin X 2017 Characterization of a polycyclic aromatic ring-hydroxylation dioxygenase from *Mycobacterium* sp. NJS-P. Chemosphere 185:67–74. doi:10.1016/j.chemosphere.2017.07.001. 28686888

[13] Kweon O, Kim S-J, Baek S, Chae J-C, Adjei MD, Baek D-H, Kim Y-C, Cerniglia CE 2008 A new classification system for bacterial Rieske non-heme iron aromatic ring-hydroxylating oxygenases. BMC Biochem 9:11. doi:10.1186/1471-2091-9-11. 18387195 PMC2358900

[14] Stingley RL, Brezna B, Khan AA, Cerniglia CE 2004 Novel organization of genes in a phthalate degradation operon of *Mycobacterium vanbaalenii* PYR-1. Microbiology 150:3749–3761. doi:10.1099/mic.0.27263-0. 15528661

[15] Habe H, Chung J-S, Ishida A, Kasuga K, Ide K, Takemura T, Nojiri H, Yamane H, Omori T 2005 The fluorene catabolic linear plasmid in *Terrabacter* sp. strain DBF63 carries the β-ketoadipate pathway genes, *pcaRHGBDCFIJ*, also found in proteobacteria. Microbiology 151:3713–3722. doi:10.1099/mic.0.28215-0. 16272392

[16] Kim S-J, Kweon O, Jones RC, Freeman JP, Edmondson RD, Cerniglia CE 2007 Complete and integrated pyrene degradation pathway in *Mycobacterium vanbaalenii* PYR-1 based on systems biology. J Bacteriol 189:464–472. doi:10.1128/JB.01310-06. 17085566 PMC1797382

[17] van Herwijnen R, Springael D, Slot P, Govers HAJ, Parsons JR 2003 Degradation of anthracene by *Mycobacterium* sp. strain LB501T proceeds via a novel pathway, through o-phthalic acid. Appl Environ Microbiol 69:186–190. doi:10.1128/AEM.69.1.186-190.2003. 12513994 PMC152392

[18] Scott A 2020 Investors chase sustainable aromatics. Chem Eng News 98:18–19.

[19] Weiss R, Guebitz GM, Pellis A, Nyanhongo GS 2020 Harnessing the power of enzymes for tailoring and valorizing lignin. Trends Biotechnol 38:1215–1231. doi:10.1016/j.tibtech.2020.03.010. 32423726

